# The Book World of Medicine and Science

**Published:** 1896-07-11

**Authors:** 


### The Book World of Medicine and Science.

Elementary Human Physiology. By John Gray McKendrick, M.D., F.R.S., with 164 Woodcuts. (London and Edinburgh: W. and R. Chambers, Limited. 1896.)

This little work has been arranged and designed to suit the syllabus of the Department of Science and Art as far as the first or elementary stage. Necessarily most of the text is occupied with explanations and descriptions of the anatomy of the body; that which deals with microscopy and function constitutes but a small part of the book. This is exactly what is wanted in a text book written for schools and elementary classes. Teachers obtaining their lecture notes from more advanced text books are apt to select the more interesting details of pure physiology and to leave the alphabet of the science or the anatomical description imperfectly understood. Two admirable features in Dr. McKendrick's little book are: (1) The introduction of a number of questions at the end of the chapters to test the knowledge of the reader; (2) the division and subdivision of the various subjects into numerous sections, with heading in diverse type, so arranged as to attract the attention and imprint themselves upon the memory of the reader. The exact features in an inexact science have been definitely, almost dogmatically, stated. The controversial points have been omitted. The student may, in fact, complete his task as far as this work is concerned and believe that physiology can explain all the functions of the body on chemical and physical grounds, instead of being, as it really is, a series of admissions of fairly profound ignorance. It is decidedly a student's book—far less dry than most of its predecessors—and well condensed although never obscure.

Health and Condition in the Active and the Sedentary. By N. E. Yorke-Davies, L.R.C.P. (London: Sampson Low, Marston, and Co. Third edition. 1895.)

This book is a work of art. It cannot exactly be called a book on diet, for it does not tell very much about diet itself. Its theme is rather the praise of the dietetic treatment of various morbid conditions. First among these comes obesity. "Of all the diseased conditions due to excess of or improper foods and want of exercise, there is not one that leads to so much discomfort or is so disastrous in its effects as corpulence." With this many will be found to agree; and, in fact, there is a great deal throughout the book which it is quite in accord with modern physiological knowledge, although, perhaps, the pathology may be of a somewhat narrow type. All through its pages, however, there runs a pleasant vein of hope. However unwieldy be the gross fatness of the hapless patient, hope is instilled into his ponderous breast; and, although some may object to such a use of the word art, we cannot but think there is something truly artistic in the use of words in such a form that, without anything offensive, and in fact while abusing quacks and quackery in unmeasured terms, hope is offered to the fat, and the way to Harley Street and health is pointed out. "From whatever cause," says the author, "the obesity may arise, or however advanced it may be, it is happily, by proper dieting for a short time, within the reach of complete and permanent cure. I think I may speak from large experience on this point, as undoubtedly within the last four or five years I have advised some thousands of victims with unvarying success, without hardship or starving, if for a time they will live by rule." Happy doctor, to have unvarying success! Of course the treatment spoken of with such confidence is to a large extent a war against starch and sugar, and there is no doubt that anyone who will take the trouble to think out the details will be able to devise a non-fat-forming diet which shall be palatable and moderately satisfying. From a medical point of view it is interesting to find one who supposes that he has "treated more cases of corpulence than anyone else in this country" maintaining so strongly as he does the relation between corpulence, gout, and rheumatism, and also the advantages of a meat diet in gout. The book is nicely written, well printed, and treats of many ailments besides obesity; in fact of most ailments, and for all of them there is hope. The skill of the writer is shown throughout its pages by the way in which sufferers from these various maladies are led to see that there is hope and to feel confidence in the powers of the dietetic specialist. Herein is art.

The Spas and Mineral Waters of Europe. By Hermann Weber, M.D., and F. Parkes Webber, M.D. (Smith, Elder, and Co. 1896. 8vo., pp. 380. 6s.)

It is to be feared that the study of balneo-therapeutics is not a favourite one with English medical men, and the cause of this neglect is not far to seek. The subject is scarcely taught in our schools or mentioned in our text-books, and but few of our fellow practitioners have time or inclination to visit the various spas and personally investigate the results of the treatment. This neglect and ignorance are much to be regretted, for it has been demonstrated often enough that much benefit accrues from a residence at one or other of the famous health resorts in such diseases as gout, rheumatism, anæmia, syphilis, diabetes, and heart affections. The book named at the head of this notice comes as a welcome addition to the English literature of the subject. It is extremely well arranged, and constitutes a most useful and reliable guide to the various spas of Europe. Each place is briefly but lucidly described, an analysis of the waters is given, the diseases for which it is suited are mentioned, and the best routes from England are given. The first five chapters are devoted to a general description of balneo-therapeutics, to the action and classification of mineral waters, to the influence of change of air and diet in assisting the water cure, and to the daily routine of life at the spas. We should not, perhaps, like to endorse the authors' views with reference to the reduction of fluids to be consumed in chronic heart diseases. No doubt all fluids taken must pass through the heart, and so throw on this organ an extra strain; but this effect is far more than counter-balanced by the good which results from the solution and excretion of poisonous matters accumulating in the capillaries. At all events, the addition of a pint or so of pure water per diem will often relieve the more urgent symptoms in chronic heart disease. The volume is well up to date, and ends with a long list of the books, pamphlets, and articles which have appeared on the subject in this and other countries.

